# 

**Published:** 1887-12-24

**Authors:** 


PRICE ONE PENNY.

[illegible]o. 65, Vol. III—December 24th, 1887.

[Registered at the General Post Office as a Newspaper.

Per Annum, 6s. 6d., post free.

Monthly Parts, 6d.; post free, 7½d.

Ditto per Ann., 7s. 6d. post free.

